# Ticks (*Argasidae* and *Ixodidae*) in Tehran Province, Central Iran: Distribution and Seasonal Activity

**DOI:** 10.1155/vmi/7844575

**Published:** 2025-06-27

**Authors:** Mojtaba Beik-Mohammadi, Maryam Tavassoli, Zakkyeh Telmadarraiy, Hassan Vatandoost, Farrokh Dabiri

**Affiliations:** ^1^Department of Medical Entomology and Vector Control, School of Public Health, Urmia University of Medical Sciences, Urmia, Iran; ^2^Health Deputy, Iran University of Medical Sciences, Tehran, Iran; ^3^Department of Medical Entomology and Vector Control, School of Public Health, Tehran University of Medical Sciences, Tehran, Iran; ^4^Rahyan Novin Danesh (RND) Private University, Sari, Mazandaran, Iran; ^5^Social Determinants of Health, Research Center, Urmia University of Medical Sciences, Urmia, Iran

**Keywords:** Acari, *Argasidae*, fauna, *Ixodidae*, ticks

## Abstract

Ticks are obligatory ectoparasites of vertebrates and can transmit pathogens, including: arboviruses, bacteria, and parasites to humans and animals worldwide. This study aimed to identify the tick species found on semi-domestic hosts in Tehran Province. This cross-sectional study, conducted from 2020 to 2021, aimed. Of 1165 captured hosts, 16.3% (*n* = 190) were infested with ticks. A total of 574 ticks were collected. Hard ticks (Acari: *Ixodidae*) comprised 86% (*n* = 493) and included 11 species, while soft ticks (Acari: *Argasidae*) made up 14% (*n* = 81) and included 3 species. The brown dog tick, *Rhipicephalus sanguineus sensu lato,* and North American tick with cattle tick, *R. annulatus* (formerly belonged to the genus *Boophilus* as *B. annulatus*), represented the highest and lowest frequencies of 39.3% and 0.3%, respectively. Notably, *Haemaphysalis inermis* was identified for the first time in Shemiranat County. The highest tick infestation intensity was observed in the Varamin district.

## 1. Introduction

Ticks are obligatory blood-feeding ectoparasites that primarily parasitize wild terrestrial vertebrates, and making them of significant zoonotic medical and veterinary importance [1]. Of the three recognized tick families: *Ixodidae, Argasidae*, and *Nuttalliellidae* (the latter being a monotypic family representing the most primitive living lineage of ticks), *Ixodidae* and *Argasidae* are the most widely recognized. To date, 896 tick species have been identified: 193 *Argasidae* and 702 *Ixodidae*. The *Nuttalliellidae* is monotypic, containing the single species *Nuttalliella namaqua *[2]. Ticks are major vectors of disease in humans and livestock, transmitting viral, bacterial, and parasitic pathogens that cause direct harm and pose significant health risks [3]. In humans, tick-borne diseases include rickettsiosis, Crimean-Congo hemorrhagic fever (CCHF), Lyme disease, tularemia, endemic relapsing fever, and anaplasmosis. Pathogens are transmitted through transovarial or transtadial mechanisms and can also cause allergic reactions, toxic effects, and irreversible paralysis [4]. Effective control of tick-borne zoonoses is crucial for both livestock health and dairy production [5]. The distribution, abundance, and species composition of ticks are critical factors in disease epidemiology and the development of effective control strategies in specific regions [6, 7]. Louis Delpy (1936) pioneered tick research in Iran [8], and early researchers emphasized the role of *Ornithodoros* ticks in transmitting *Borrelia* within the country [9]. Numerous tick studies have been conducted across various Iranian regions, including the southwest (Sistan and Baluchestan), northwest (West Azerbaijan and Ardebil provinces), north (Gilan, Golestan, Mazandaran, and Qazvin provinces), south (Hormozgan province), and west (Kurdistan and Kohgilouyeh provinces) [10–15]. Some studies have focused on compiling lists of adult ticks collected from domestic animals, with particular emphasis on ixodid ticks, which transmit several dangerous diseases to goats and wild sheep [16]. Yakhchali et al. [17] also explored the distribution and diversity of ixodid ticks in northern and southern Iran [17–19]. In recent years, factors such as global warming, illegal cross-border livestock movement, drought, and groundwater depletion have contributed to increased animal movement to more fertile pastures and consequent changes in tick populations in Iran. Given the impact of climate change, updated information on the abundance, distribution, and diversity of tick genera and species in different regions of the country is increasingly needed. This study aims to address this gap by investigating the biodiversity and fauna of ticks infesting the most important domestic and peridomestic mammals and birds in Tehran Province, a key economic and administrative region of Iran.

## 2. Materials and Methods

### 2.1. Study Area

This research was conducted in Tehran Province, Iran, located between 34° and 36.5°N latitude and 50° and 53°E longitude. The province is bordered by Mazandaran Province to the north, Qom Province to the south, Markazi Province to the southwest, Alborz Province to the west, and Semnan Province to the east. The average elevation of Tehran is 1200 m above sea level. Tehran has a temperate climate in its mountainous regions and a semi-arid climate in its plains, situated between climatic and oceanic plates.

### 2.2. The Sample Size

The sample size was calculated using the following formula, with parameters *d* = 0.045, *p* = 0.3, and (1 − p) = 0.7 [19, 20]. At a 95% confidence level, the minimum required sample size was determined to be 400 ticks.

### 2.3. The Selection of the Study Areas

The climate of Tehran Province can be divided into two main types: mountainous areas, which have a temperate to cold climate (characterized by wider temperature ranges and more distinct seasonal variations), and plains, which have a semi-desert climate. These semi-desert regions receive less precipitation than the potential evapotranspiration but are not as arid as true desert climates. Firoozkooh, Damavand, and Shemiranat are located within the mountainous climatic zone, while most cities in Tehran Province, such as Varamin, Pakdasht, Shahriyar, Shahr-e Rey, Islamshahr, and Robat Karim, are situated in the plains. A large proportion of ticks were collected from 30 carefully selected villages representing the climatic diversity of Tehran Province, including 8 villages in the mountainous region and 22 in the plains. Livestock distribution and population data were obtained from the National Veterinary Organization. Based on these ecological data, indoor and outdoor tick sampling was conducted at various sites throughout the year. A total of 1165 animals were examined, including a variety of domestic and peridomestic mammals and birds: cattle (*Bos taurus*), sheep (*Ovis aries*), dogs (*Canis lupus familiaris*), camels (*Camelus dromedarius*), chickens (*Gallus gallus domesticus*), and pigeons (*Columba livia*).

### 2.4. Sample Collection and Preparation

**Figure 1:**
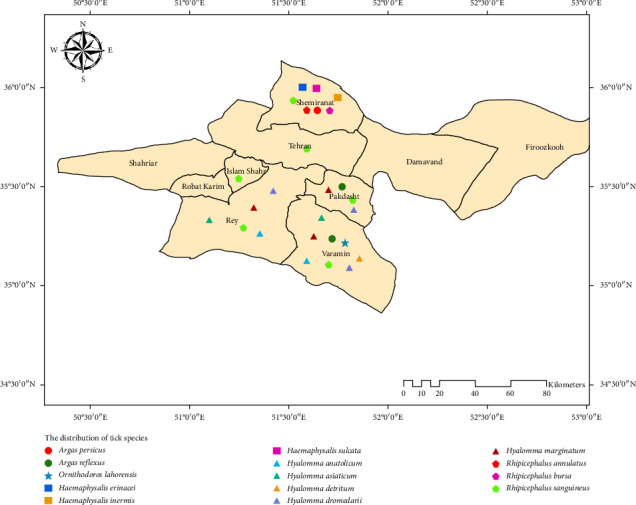
The distribution of tick species in Tehran Province, Iran, April to February 2021.

From spring to the end of winter 2021, a cross-sectional study was conducted across various areas of Tehran Province to investigate tick distribution and fauna. Several sampling steps were followed at each study site. Samples were collected from both industrial and conventional livestock pens. Ticks were carefully removed from hosts using fine-tipped curved forceps, primarily from areas such as the earlobes, groin, tail base, and back (Figure 1). For poultry, the groin, abdomen, and underwings were thoroughly examined. To ensure accurate identification, the ticks' mouthparts were carefully preserved during collection. Collected ticks were placed in tubes, transferred to a flask, and transported to the Medical Entomology Laboratory at Urmia University of Medical Sciences, School of Health, where they were stored at 22°C. Tick identification was performed using standard diagnostic keys [21–23].

## 3. Results

**Figure 2:**
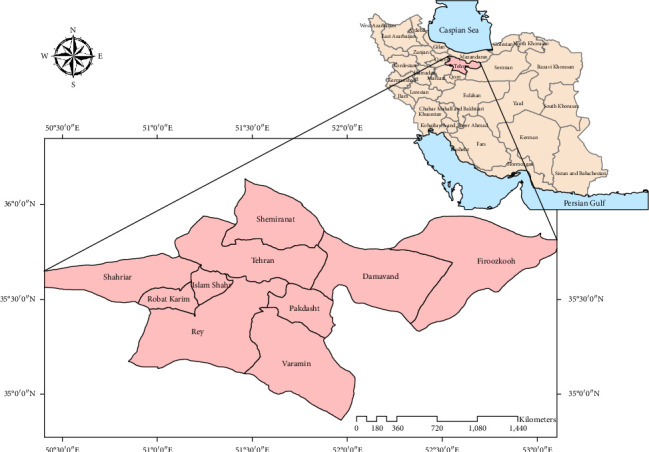
Location of sampling: Tehran Province, Iran, April to February, 2021.

A year-long study, from spring to winter 2021, investigated the fauna and seasonal activity of ticks belonging to the *Argasidae* and *Ixodidae* families in Tehran Province. Sampling was conducted in two ecological zones (plains and mountains) across 30 selected villages (Figure 2 and Table 1). A total of 1165 hosts were examined, including cattle, sheep, dogs, camels, chickens, and pigeons. Analysis of livestock infestation severity in different ecological areas revealed that 34.6% of the studied livestock were from the mountainous region, while 65.4% were from the plains. Overall, 16.3% of the livestock were infested with ticks, with 36.9% of the infested hosts originating from the mountainous region and 63.1% from the plains. Of the 574 ticks collected (see correction below), 43.4% were from the mountainous region and 56.6% were from the plains (Table 2). Among the 16.3% of infested hosts (*n* = 190), a total of 574 ticks were collected. The ticks were identified as belonging to both the *Argasidae* and *Ixodidae* families: 14.1% (*n* = 81) were from the *Argasidae* family, and 85.9% (*n* = 493) were from the *Ixodidae* family (Table 3). Among the five identified genera (Table 4), *Rhipicephalus* had the highest frequency (40.6%), while *Ornithodoros* had the lowest (2.3%). As shown in Table 5, among the 14 identified tick species, the most and least abundant were *Rhipicephalus sanguineus* and *Rhipicephalus annulatus* (*Ixodidae*), and *Argas persicus* and *Argas reflexus* (*Argasidae*), respectively. Figure 1 illustrates the distribution of these species across different regions of Tehran Province. Based on the percentage abundance of tick species collected in mountainous and plain climates, several distinct distributions were observed. *Argas persicus*, *Rhipicephalus bursa, R. annulatus* and all *Haemaphysalis* species (*Haemaphysalis sulcata*, *Haemaphysalis inermis*, *H. erinacei*) were found exclusively in mountainous climates. Conversely, *Ornithodoros lahorensis*, *Argas reflexus*, and all *Hyalomma* species (*Hy. marginatum*, *Hy. asiaticum*, *Hy. dromedarii*, *Hy. anatolicum*, and *Hy. scupense*) were found only in plain climates. *R. sanguineus* was the only species found in both climate types. *R. sanguineus* also exhibited the highest sex ratio in both mountainous and plain regions (Table 6). Regarding tick infestation, the majority of ticks were collected from sheep (59%), while the fewest were collected from cow (0.5%). Among the ticks collected in the *Ixodidae* family, *R. annulatus* was found on cow, *Haemaphysalis* was found only on sheep and goats, and *Rhipicephalus* and *Hyalomma* were found on all hosts except pigeons, chickens, and cage walls. Ticks from the Argasidae family, specifically *Ornithodoros*, were found on cage walls, while the genus *Argas* was found on both pigeon and chicken hosts. No soft ticks were collected from cow, sheep, goats, camels, or dogs (Table 7). The highest rate of tick infestation in domestic livestock occurred in the spring, while the lowest rate was observed in the winter. The rate of tick infestation in different seasons is as follows: In the spring, all species were found (except for *R. annulatus*, *O. lahorensis* and *Hy. scupense*. In the summer, *Rhipicephalus* and *Hyalomma* were present. In autumn, *Hyalomma* was dominant, and in winter, the genera *Argas* and *Ornithodoros* (family *Argasidae*) were most prevalent (Table 8).

## 4. Discussion

There are few studies on tick fauna and pathogen transmission in Tehran Province [19]. Five genera (*Argas, Ornithodoros, Hyalomma, Haemaphysalis,* and *Rhipicephalus*) were detected in two studies conducted in Boyer-Ahmad and Sanandaj counties, differing from our study due to the absence of *R. annulatus* [10, 24]. Sofizadeh et al. [8] reported six genera and 15 species from the Argasidae and Ixodidae families in Golestan Province. The key differences between their study and ours are the presence of *Ixodes ricinus* in their samples and the detection of *Argas reflexus* and *Ornithodoros lahorensis* in ours. Our study identified a total of five genera and 14 species. The identification of four common tick genera (*Argas*, *Hyalomma*, *Haemaphysalis*, and *Rhipicephalus*) in both the Golestan Province study and our study is likely due to the comparable climates of Tehran and Golestan provinces, given their geographic proximity. In this study, *Haemaphysalis inermis* was reported for the first time in Tehran Province. Previously, *H. inermis* has been observed in several other Iranian provinces, including West Azerbaijan, East Azerbaijan, Gilan, Mazandaran, Golestan, Razavi Khorasan, South Khorasan, Kurdistan, Ilam, and Lorestan [25–28]. *H. inermis* has been noted for causing tick paralysis. In Poland, it is suspected to be a vector for tick-borne encephalitis (TBE) virus [29]. In a study conducted in the Zahedan district of Sistan and Baluchestan Province (southeastern Iran), *H. inermis* was confirmed as a vector for CCHF virus [30]. The unexpected presence of this species in Tehran Province prompted repeated samplings and further surveys, ultimately confirming its presence in sheep and goats. The reasons for this occurrence, including legal livestock displacements and trafficking, remain unclear. However, the presence of this species should be considered by future researchers studying tick-borne disease epidemiology in the region, particularly given that Tehran County, the capital of Iran, is located in this province and plays a central role in social, economic, political, and administrative matters. In two studies from West Azerbaijan Province, seven genera were present, including *Argas, Ornithodoros, Hyalomma, Haemaphysalis, Rhipicephalus, Dermacentor,* and *Boophilus*. Our study differed in the absence of *Dermacentor* [9, 31]. One possible reason for this is that *Dermacentor* ticks have difficulty adapting to low altitudes above sea level. Additionally, this genus has a limited distribution in the country. A previous study conducted between 2002 and 2005 found *Dermacentor* only in Kurdistan, Ardabil, East Azerbaijan, Zanjan, Khorasan, and Semnan provinces [32]. No *Ixodes* genera were found in our study. This could be because *Ixodes* are common in Iran's northern provinces, such as Gilan, Mazandaran, and Golestan [33]. However, the high Alborz mountain range between Tehran Province and these northern regions may limit the movement of this genus. Additionally, *Ixodes* ticks thrive in cold and wet climates, and Tehran Province, compared to the northern provinces, has relatively lower humidity. Thus, the absence of *Ixodes* in our study area may be linked to lower humidity levels. *Argas* ticks from the Argasidae family were found in Shemiranat, a mountainous city in northern Tehran Province, at a rate of 10.8%. Abbasian and Mazlum previously noted the spread of this tick throughout Iran, except in the deserts and coastal areas along the Persian Gulf and the Caspian Sea [26, 34]. The autumn season had the highest frequency of tick infestation, which aligns with findings from Boyer-Ahmad County in the south and Sanandaj and Bijar counties in Kurdistan Province (northwest Iran) [10, 24, 35]. This contrasts with other studies conducted in West Azerbaijan [9, 31]. *Argas reflexus* was collected in Pakdasht, a plain climate area, during spring and winter but was absent in summer and autumn. This finding contradicts the results of Rafinejad et al. [35]; who did not collect this species in spring or winter in Bijar city. One possible explanation for the low abundance of this species in Tehran Province is the limited practice of pigeon keeping and hunting in most villages. *Ornithodoros lahorensis* is prevalent throughout Iran, except in Golestan, Gorgan, and Khuzestan [36]. However, some studies have failed to detect this species in Sistan and Baluchestan or Khuzestan [26]. In our study, this species was found only in winter in Varamin's plain climate, specifically in cages and stalls. This finding is consistent with previous results from Hamedan [18]. A possible reason for the abundance of *O. lahorensis* during the cold season is that livestock are housed in stalls during winter, where these ticks, often in their nymphal stage, are more commonly found within the animals' wool. Other species of the genus *Ornithodoros*, such as *O. tholozani*, *O. canestrinii*, *O. erraticus*, and *O. tartakovskyi*, were absent in this study. A likely reason for their absence is the sanitary improvement of livestock enclosures. Many of these enclosures have been upgraded with cement block walls, preventing ticks from burrowing into the walls to rest and hide. Additionally, *Ornithodoros* ticks are rarely found in rodent nests, and *O. erraticus* may be more easily collected from such nests [18, 26, 36]. *Haemaphysalis sulcata* was the most abundant *Haemaphysalis* species in our study, found in Shemiranat (a mountainous climate). This species is typically found in cold regions at altitudes of around 2000 m above sea level. It was collected only in spring, whereas Sofizadeh et al. [8] reported low abundance during both autumn and winter in Golestan Province. *H*. *erinacei*, like the other two *Haemaphysalis* species in this study, was also found in the mountainous city of Shemiranat. *R. annulatus* had the lowest frequency (0.35%) in Tehran Province and was detected only in cattle in Shemiranat, a city with a mountainous climate similar to the northern provinces of Iran. In our study, *R*. *sanguineus* was the dominant species, consistent with findings from many other researchers [8, 37–39]. *R*. *bursa* was found in Shemiranat, collected from sheep during the spring and summer seasons, aligning with a study conducted in Amol, northern Iran [33]. The absence of *R. bursa* in autumn and winter can be attributed to its need for high humidity and temperature for its life cycle. This species has been collected from various locations, including Tehran, Islamshahr, Shemiranat, Shahr-e Ray, Pakdasht, and Varamin, spanning both plain and mountain climates, consistent with the findings of Nasibeh et al. [37] in Ghaemshahr, Mazandaran Province. *R. bursa* requires at least three hosts during its life cycle. In our study, the host diversity for this species included sheep, goats, camels, and dogs, but it was never found on cattle. This absence in cattle is likely due to farmers' heightened sensitivity in managing ectoparasites on this host, driven by both economic considerations and the high vulnerability of cattle. Seasonal activity of *R. bursa* occurred throughout the year, with maximum activity in spring and minimum activity in winter, consistent with the findings of Sofizadeh et al. [40] in Golestan Province. *H*. *marginatum* was the second most common tick species after *R. sanguineus* in our study. This species was found on multiple hosts, including sheep, camels, and cattle, in all seasons across Pakdasht, Varamin, and Shahr-e Rey. Its peak activity was observed in spring and summer, with minimal activity in autumn and winter, a trend similar to the results of Tavakoli [41] in Lorestan Province. *Hy. scupense* was also collected, albeit in low numbers, primarily from camels in Varamin (a plain region with a hot and dry climate). Iran's diverse mountainous, desert, and forested regions, with varying tropical, subtropical, and temperate climates, along with differences in tick control methods, result in varying dominant tick species across regions. Therefore, understanding the species composition, frequency, and distribution in different areas is crucial for understanding the epidemiology of tick-borne diseases and implementing effective control measures. The infestation of cattle, sheep, goats, and birds with ticks, and their role in transmitting pathogens to humans, underscores the importance of tick management. Health officials should prioritize tick control efforts by educating ranchers on the significance of ticks, promoting proper livestock hygiene, eliminating tick habitats, and encouraging the use of appropriate treatments such as dips and drainage systems.

## 5. Conclusion

*Haemaphysalis inermis* has been reported for the first time in Shemiranat, a city in Tehran Province. The highest and lowest tick distributions were observed during spring and winter, respectively. Understanding these seasonal variations in tick activity is essential for developing effective disease management strategies.

## Figures and Tables

**Table 1:** Selected villages from two zones, Tehran Province, Iran, April to February, 2021.

	Plains villages	Mountain villages
Tochāl		
	Qeshlaq-e Qaleh Now	Hashemak
	Mohammad Abad	

Abdolabad-e Pain		
	Qermez Tappeh	Meygon
	Jitou	

Karimabad		
	Shurabad	Kond Olia-Sofla
	Bazare sang	

Eslamshahr Railway Station		
	Kahrizak	Oshan
	Abbasabad	

Mohammadabad-e Arab		
	Quheh	Roodak
	Khatunabad	

Aminabad		
	Sharifabad	Barg-e Jahan
	Varamin Canal Road	

Kohanz		
	Alvard	Dehnār
	Ferunabad	

**Table 2:** The infectivity rates of ticks' existence among investigated livestock were divided into different climate zones (Tehran Province, Iran).

Climate	Number of animals examined (%)	Number of infected livestock (%)	Number of ticks collected (%)
Mountain	(6/34) 403	(85/36) 70	(38/43) 249
Plain	(4/65) 762	(15/63) 120	(62/56) 325
Totality	1165 (100)	190 (100)	574 (100)

**Table 3:** The number and frequency of tick families were collected in Tehran Province, Iran, from April to February 2021.

Family	Number/frequency (%)
*Ixodidae*	231 (40.24)
	221 (38.5)
	39 (6.8)
	2 (0.35)

*Argasidae*	68 (11.85)
	13 (2.26)

Totality	574 (100)

**Table 4:** Genera of *Argasidae* and *Ixodidae* in Tehran province, Iran, April to February, 2021.

Genera	Number/frequency (%)
*Rhipicephalus*	231 (40.24)
*Hyalomma*	221 (38.5)
*Haemaphysalis*	39 (6.8)
*Boophilus*	2 (0.35)
*Argas*	68 (11.85)
*Ornithodoros*	13 (2.26)
Totality	574 (100)

**Table 5:** The identified tick species were collected in Tehran Province, Iran, from April to February 2021.

Species of ticks	Frequency (%)
*Argas persicus*	10.8
*Argas reflexus*	1.05
*Ornithodoros lahorensis*	2.26
*R.* (*syn. Boophilus*) *annulatus*	0.35
*Rhipicephalus bursa*	0.87
*Rhipicephalus sanguineus*	39.38
*Haemaphysalis sulcata*	4.0
*Haemaphysalis inermis*	1.75
*Haemaphysalis erinacei*	1.05
*Hyalomma marginatum*	20.38
*Hyalomma asiaticum*	8.54
*Hyalomma dromedarii*	6.62
*Hyalomma anatolicum*	2.26
*Hy. scupense* (syn. Hy. detritum)	0.69
	Total: 100

**Table 6:** The percent of collected ticks' species related to topographical zone types, Tehran Province, Iran.

Tick species	Mountain		Plain	
	Number	(%)	Number	(%)
*Rhipicephalus sanguineus*	141	21/63	85	79/36
*Hyalomma marginatum*	0	0	117	100
*Argas persicus*	62	100	0	0
*Hyalomma asiaticum*	0	0	49	100
*Hyalomma dromedarii*	0	0	38	100
*Haemaphysalis sulcata*	23	100	0	0
*Hyalomma anatolicum*	0	0	13	100
*Ornithodoros lahorensis*	0	0	13	100
*Haemaphysalis inermis*	10	100	0	0
*Haemaphysalis erinacei*	6	100	0	0
*Argas reflexus*	0	0	6	100
*Rhipicephalus bursa*	5	100	0	0
*Hyalomma scupense (syn. Hy. detritum)*	0	0	4	100
*Rhipicephalus. (syn. Boophilus) annulatus*	2	100	0	0
Totality	249	43.38	325	56.62

**Table 7:** The frequency of tick species by host type in Tehran Province, Iran.

Tick species	Hosts							Barn wall
	Cow	Sheep	Goat	Camel	Dog	Pigeon	Chicken	
*Rhipicephalus sanguineus*	0	176	30	3	17	0	0	0
*Hyalomma marginatum*	1	65	0	51	0	0	0	0
*Argas persicus*	0	0	0	0	0	0	62	0
*Hyalomma asiaticum*	0	45	0	4	0	0	0	0
*Hyalomma dromedarii*	0	10	6	22	0	0	0	0
*Haemaphysalis sulcata*	0	21	2	0	0	0	0	0
*Hyalomma anatolicum*	0	7	0	6	0	0	0	0
*Ornithodoros lahorensis*	0	0	0	0	0	0	0	13
*Haemaphysalis inermis*	0	8	2	0	0	0	0	0
*Haemaphysalis erinacei*	0	4	2	0	0	0	0	0
*Argas reflexus*	0	0	0	0	0	6	0	0
*Rhipicephalus bursa*	0	3	2	0	0	0	0	0
*Hyalomma scupense (syn. Hy. detritum)*	0	0	0	4	0	0	0	0
*Rhipicephalus. (syn. Boophilus) annulatus*	2	0	0	0	0	0	0	0
Totality	3	339	44	90	17	6	62	13

**Table 8:** Identify and determine tick distribution based on seasonal distribution in Tehran Province, Iran.

Tick species	Season				Total	(%)
	Spring	Summer	Autumn	Winter		
*Rhipicephalus sanguineus*	197	14	12	3	211	38/39
*Hyalomma marginatum*	61	44	7	5	117	38/20
*Argas persicus*	10	0	30	22	62	8/10
*Hyalomma asiaticum*	22	8	11	8	49	54/8
*Hyalomma dromedarii*	7	15	10	6	38	62/6
*Haemaphysalis sulcata*	23	0	0	0	23	4
*Hyalomma anatolicum*	7	3	3	0	13	26/2
*Ornithodoros lahorensis*	0	0	0	13	13	26/2
*Haemaphysalis inermis*	10	0	0	0	10	75/1
*Haemaphysalis erinacei*	6	0	0	0	6	05/1
*Argas reflexus*	3	0	0	3	6	05/1
*Rhipicephalus bursa*	3	2	0	0	5	87/0
*Hyalomma scupense (syn. Hy. detritum)*	0	0	4	0	4	69/0
*Rhipicephalus. (syn. Boophilus) annulatus*	0	0	2	0	2	35/0
Totality	349 (11 species)	86 (6 species)	78 (8 species)	60 (7 species)	574 (14 species)	100

## Data Availability

The specimens mentioned in this article are deposited in the Medical Entomology Laboratory at Urmia University of Medical Sciences, School of Health. All other data are provided within the manuscript.
